# Expression of MTA1 promotes motility and invasiveness of PANC-1 pancreatic carcinoma cells

**DOI:** 10.1038/sj.bjc.6601535

**Published:** 2004-01-20

**Authors:** M D Hofer, A Menke, F Genze, P Gierschik, K Giehl

**Affiliations:** 1 Department of Pharmacology and Toxicology, University of Ulm, 89069 Ulm, Germany; 2 Department of Internal Medicine, University of Ulm, 89069 Ulm, Germany

**Keywords:** MTA1, pancreatic carcinoma, migration, invasion and metastasis

## Abstract

The human metastasis-associated protein 1 (MTA1) is a constituent of the nucleosome-remodelling and -deacetylation complex. Its expression has been correlated with the invasion and metastasis of epithelial neoplasms. To address the functional consequences of MTA1 expression in pancreatic carcinoma cells, we have established PANC-1 pancreatic carcinoma cells that stably express MTA1 as an enhanced green fluorescent fusion protein (EGFP–MTA1). Here, we demonstrate that heterologous expression of EGFP–MTA1 markedly enhanced the cellular motility and the invasive penetration of epithelial barriers by the cells. Expression of EGFP-MTA1 had no effect on substrate-independent growth, but reduced substrate-dependent cell proliferation. In addition, the organisation of the cytokeratin filament system and the localisation of the actin cytoskeleton-associated protein IQGAP1 were distinctly altered in EGFP–MTA1-expressing cells. These results indicate that enhanced expression of MTA1 promotes the acquisition of an invasive, metastatic phenotype, and thus enhances the malignancy of pancreatic adenocarcinoma cells by modulation of the cytoskeleton.

The metastasis-associated gene 1 (*mta1*) was identified by differential cDNA screening using cell lines derived from highly metastatic mammary adenocarcinomas (Toh *et al*, 1994), and expression of metastasis-associated protein 1 (MTA1) correlated with the invasive and metastatic potential of cultured mammary tumour cells (Toh *et al*, 1994, 1995). Enhanced expression of MTA1 mRNA was also found in a variety of other human cancerous tissues and carcinoma cell lines, for example in colorectal, gastric (Toh *et al*, 1997), or oesophageal (Toh *et al*, 1999) carcinomas and thymoma (Sasaki *et al*, 2001). Comparative analyses revealed that MTA1 mRNA was differentially expressed in nonmetastatic and metastatic tumours, thus pointing towards a role of MTA1 in enhancing the metastatic potential of malignant tumour cells (reviewed in Nicolson *et al*, 2003).

The MTA1 protein is a representative of the MTA1 family highly conserved throughout evolution with 88% identities of nucleotide and 96% identities of amino-acid sequences between rat and human MTA1. Other members of this protein family are MTA1-L1 (Futamura *et al*, 1999), MTA2 (Zhang *et al*, 1999), and MTA3 (Simpson *et al*, 2001). Homologues of MTA1 and MTA2 were found in *Caenorhabditis elegans* and *Drosophila* (Herman *et al*, 1999; Solari *et al*, 1999; Erkner *et al*, 2002).

The *mta1* gene has been mapped to the centre of a 1.6 Mb region of human chromosome 14q 31.2 (Martin *et al*, 2001) and encodes a protein of 715 amino acids with a calculated molecular mass of 80.8 kDa (Toh *et al*, 1994; Nawa *et al*, 2000). Sequence analysis revealed that the human MTA1 protein contains a proline-rich region (aa: 697–705), which matches the consensus sequence for the src homology 3 domain (SH3)-binding site (Toh *et al*, 1995; Nawa *et al*, 2000). A second potential SH3-binding site is located between amino acids 545 and 552. Other potential protein–protein and protein–DNA interaction domains are: a putative GATA-like zinc-finger-motif, a bromo-adjacent-homology domain (BAH domain), a SWI3, ADA2, N-CoR, and TFIIIB DNA-binding domain (SANT domain), several putative phosphorylation sites for tyrosine kinases, protein kinase C, and casein kinase 2, and three putative nuclear localisation sequences (Nawa *et al*, 2000; reviewed in Nicolson *et al*, 2003). Although MTA1 and the related protein MTA2 were recently identified as constituents of the nucleosome-remodelling and deacetylation complex (NuRD) (Xue *et al*, 1998; Zhang *et al*, 1999), the exact role of MTA proteins in the NuRD complex, especially in chromatin remodelling and transcriptional repression, is unknown. Mazumdar *et al* (2001) have reported that activation of the heregulin/human epidermal growth-factor receptor 2 (HER2) signalling pathway in oestrogen-receptor-positive breast cancer cells stimulated MTA1 expression. The augmented expression contributes to enhanced histone deacetylase activity, inhibition of oestrogen-receptor element-driven gene transcription, and progression to an invasive phenotype. Moreover, it has been reported that MAT1 (ménage á trios 1), an assembly/targeting factor for cyclin-dependent kinase-activating kinase (CAK), is an MTA1-binding protein (Talukder *et al*, 2003). The results reported in the latter study suggest that MTA1 might inhibit CAK-induced oestrogen receptor transactivation function by recruiting histone deactylase.

Pancreatic adenocarcinoma is a highly aggressive malignant tumour, which is characterised by pronounced invasiveness and rapid progression. Although molecular pathological and genetic studies have presented a framework of the cellular alterations that are associated with pancreatic adenocarcinoma, most of the data comprise only correlative links to the underlying cancer biology (Bardeesy and DePinho, 2002). In pancreatic cancer tissues, the MTA1 mRNA expression levels seem to be correlated with lymph node metastasis, whereas in established pancreatic carcinoma cell lines MTA1 mRNA levels were not correlated with the cells' propensity to metastasise (Iguchi *et al*, 2000). Thus no definitive role of MTA1 in the progression of pancreatic cancer has yet been established.

In the present study, we have examined whether enhanced expression of MTA1 in itself modifies the malignant phenotype of pancreatic carcinoma cells. We describe that expression of an enhanced green fluorescent protein (EGFP)–MTA1 fusion protein in the human pancreatic carcinoma cell line PANC-1 resulted in nuclear localisation of the protein and caused a marked enhancement of cellular motility and invasion through epithelial barriers and, at the same time, a reduction of cell proliferation. Furthermore, MTA1-expressing PANC-1 pancreatic carcinoma cells exhibited distinct changes in arrangement and localisation of cytoskeletal proteins, such as keratins and IQGAP1. The results demonstrate that enhanced expression of MTA1 triggers the development of motile, invasive pancreatic carcinoma cells by altering the organisation of the cellular cytoskeleton.

## MATERIALS AND METHODS

### Materials

[^32^P]dCTP (5000 Ci mmol^−1^) was obtained from Amersham Biosciences (Freiburg, Germany). 4,6-Diamidino-2-phenylindole dihydrochloride (DAPI) was from Sigma (Taufkirchen, Germany). Primary antibodies: monoclonal anti-GFP (clones 7.1 and 13.1) (Roche Diagnostics, Mannheim, Germany), monoclonal anti-*β*-actin (clone AC-15) (Sigma, Taufkirchen, Germany), polyclonal anti-human pancreatic polypeptide (DakoCytomation, Hamburg, Germany), monoclonal anti-human cytokeratin 18 (DakoCytomation, Hamburg, Germany), monoclonal anti-IQGAP1 (Zymed, San Francisco, CA, USA). Secondary antibodies: peroxidase-conjugated goat anti-mouse IgG, alkaline phosphatase-conjugated anti-mouse IgG (Sigma, Taufkirchen, Germany); Cy3-conjugated goat anti-mouse (Jackson ImmunoResearch Laboratories, Hamburg, Germany). All other chemicals were of analytical grade and obtained from standard suppliers.

### Cell lines and culture conditions

Human pancreatic carcinoma cell lines PANC-1 (CRL 1469), BxPC-3 (CRL 1687), and MiaPaCa-2 (CRL 1420) were obtained from American Type Culture Collection ATCC (Manassas, VA, USA). PaTu 8988t and PaTu 8988s were kindly provided by Dr HF Kern (Marburg, Germany) (Elsasser *et al*, 1992). Cells were cultured in Dulbecco's modified Eagle's medium (DMEM) supplemented with L-glutamine (2 mM), penicillin–streptomycin (100 IU ml^−1^–0.1 mg ml^−1^), and 10% (v v^−1^) fetal calf serum. Cell culture media and supplements were from Invitrogen (Karlsruhe, Germany). Cells were incubated at 37°C in a humidified atmosphere of 90% air and 10% CO_2_. Serum starvation was performed in DMEM without supplements. Liposome-mediated DNA transfection was carried out with 30 *μ*l DMRIE-C reagent (Invitrogen, Karlsruhe, Germany) and 15 *μ*g DNA per 10 cm dish of subconfluent cells according to the manufacturer's instructions. Enhanced green fluorescent protein- and EGFP–MTA1-expressing cell clones were obtained by cultivation of cells in medium supplemented with 1.5 mg ml^−1^ G418 (Invitrogen, Karlsruhe, Germany). Selected, green fluorescent cell clones were continuously kept in this selection medium.

### Northern blot analysis

Total RNA was extracted using the RNeasy Midi Kit from Qiagen (Hilden, Germany) according to the manufacturer's instructions. For Northern blot analysis, 30 *μ*g of total RNA was separated by denaturing formaldehyde–agarose gel electrophoresis (1% (w v^−1^) agarose) and transferred onto Hybond N nylon membranes (Amersham Biosciences, Freiburg, Germany), as described in Menke *et al* (1997). Blots were hybridised with [^32^P]dCTP-labelled rat MTA1 cDNA and the amount of transferred mRNA was analysed by rehybridising blots with [^32^P]dCTP-labeled mouse *β*-actin cDNA. [^32^P]dCTP labelling was performed using the Multiprime DNA labelling system (Amersham Biosciences, Freiburg, Germany).

### Cloning of a pEGFP-C2/MTA1 expression plasmid

A pBluescript SK(−) vector containing the coding region of the rat MTA1 cDNA in the *Bam*HI restriction site was kindly provided by Dr A Nawa (University of Texas, Houston, TX, USA). The insert was ligated in frame as a *Bam*HI fragment into the pEGFP-C2 vector (Clontech BD Biosciences, Heidelberg, Germany). The sequence was verified by DNA sequencing.

### Cell lysate preparation and protein determination by immunoblot analysis

The expression of EGFP–MTA1 protein in transfected cells was analysed in cell lysates by immunoblotting. Approximately 5 × 10^6^ cells in a 10 cm dish were washed with ice-cold phosphate-buffered saline (PBS) (140 mM NaCl, 2.7 mM KCl, 8 mM Na_2_HPO_4_, 1.5 mM KH_2_PO_4_, pH 7.2), and homogenised in 500 *μ*l of RIPA buffer (50 mM Tris/HCl, pH 7.2, 150 mM NaCl, 10 mM MgCl_2_, 1% (v v^−1^) Triton X-100, 0.5% (w v^−1^) sodium deoxycholate, 0.5% (w v^−1^) SDS, 10 *μ*g ml^−1^ aprotinin, 10 *μ*g ml^−1^ leupeptin, and 0.1 mM phenylmethylsulphonyl fluoride (PMSF)) by 10 strokes in a glass–glass Dounce homogeniser. The suspension was cleared by centrifugation in a benchtop centrifuge at 15 800 **g** for 15 min at 4°C and the protein concentration was determined by the bicinchoninic acid assay (Pierce, Sankt Augustin, Germany), using bovine serum albumin fraction V (BSA; 2 mg ml^−1^) as a standard. Cell lysates (130 *μ*g of protein) were separated on 10% SDS–polyacrylamide gels and transferred onto nitrocellulose membranes (Schleicher & Schuell, Dassel, Germany). Enhanced green fluorescent proteins were detected by using anti-GFP and peroxidase-conjugated secondary antibodies, and the ECL Western Blotting Detection System (Amersham Biosciences, Freiburg, Germany). Equal loading was controlled by reprobing the blot with anti-*β*-actin and alkaline phosphatase-conjugated secondary antibodies.

### Growth and migration assays

#### Proliferation assays

For proliferation assays, 2 × 10^3^ cells were seeded per well of a 12-well cell culture dish in DMEM supplemented with 10% FCS. Every 48 h, cells of two independent wells were detached by trypsinisation and cell numbers were determined using a haemocytometer chamber. Growth curves were calculated by nonlinear regression analysis and doubling times were determined for the exponential growth phase using the GraphPad Prism 3.02 software (GraphPad Software, San Diego, USA).

#### Soft agar assays

Soft agar assays were performed as described in Weisz *et al* (1999). Briefly, for one well of a six-well plate, 1 × 10^4^ cells (in DMEM with 10% FCS) were mixed with 0.5 ml of 0.34% (w v^−1^) agar and seeded onto 1.5 ml of bottom agar containing 0.5% (w v^−1^) agar in DMEM with 10% FCS. The upper agar layer was covered with 250 *μ*l growth medium. Cells were incubated at 37°C and 10% CO_2_ and the medium was replaced every 48 h. Number and size of cell colonies were determined after incubation periods of 14 and 28 days.

#### Migration assays

To determine cell migration, 5 × 10^4^ cells were seeded in 500 *μ*l DMEM containing 10% FCS on top of noncoated polyethylene terephthalate (PET) membranes of trans-well cell culture inserts (12-well inserts, 8 *μ*m pore size; BD Biosciences, Heidelberg, Germany). The bottom chamber was filled with 1.2 ml of DMEM. After 1 h, mitomycin C (10 *μ*g ml^−1^; Sigma, Taufkirchen, Germany) was added to the medium in the upper chamber to inhibit cell proliferation. Cells were incubated for 2 h. Thereafter, cells in the upper chamber were washed with DMEM and incubated in 400 *μ*l DMEM. The medium in the bottom chamber was replaced by 1.2 ml of DMEM with or without 10% FCS. After 24 h, the cells were fixed in 4% (w v^−1^) paraformaldehyde in PBS, stained for 15 min with Harris' haematoxylin solution (Merck, Darmstadt, Germany), and washed three times for 15 min each in 2 ml each of tap water. Cells that had remained on top of the membrane were wiped away. Cells that had migrated to the bottom side of the membrane were visualised under the microscope and quantified by counting the number of cells in three randomly chosen visual fields at 100-fold magnification.

### Chorioallantoic membrane (CAM) invasion assay

The invasive potential of the cells was analysed using the CAM-model previously described by Kunzi-Rapp *et al* (2001) with some modifications. Briefly, cells (5 × 10^5^ cells in 20 *μ*l DMEM) were seeded into a silicone ring (5 mm diameter) placed on the chorioallantoic membrane of fertilised chicken eggs on day 5 of the breeding protocol and incubated for another 6 days at 37.8°C, 60% relative humidity. The chorioallantoic tissue area containing the silicone ring was isolated, fixed with 4% (v v^−1^) formaldehyde, paraffin-embedded, and 4 *μ*m sections were transferred to microscopic slides and stained with haematoxylin and eosin. For immunohistochemistry, sections were mounted on poly-L-lysine-coated slides and subsequently deparaffinised and rehydrated. Antigen retrieval was carried out by heating in vapour for 20 min in 10 mM citrate buffer, pH 6.0, and nonspecific binding sites were blocked with 10% goat serum for 20 min at room temperature. Polyclonal antiserum against pancreatic polypeptide was applied at a dilution of 1 : 100 according to the manufacturer's protocol (DakoCytomation, Hamburg, Germany). Antibodies were detected using the Histostain-Plus streptavidin peroxidase staining procedure with AEC chromogen/substrate reagent (Zymed, San Francisco, CA, USA).

### Immunofluorescence Staining

For immunofluorescence staining, 1 × 10^4^ cells were seeded on glass coverslips (13 mm diameter) and grown to about 90% confluence. After fixation with 4% (w v^−1^) paraformaldehyde in PBS, cell membranes were permeabilised for 15 min with 0.2% (v v^−1^) Triton X-100 in PBS, and nonspecific binding sites were blocked with 3% BSA (w v^−1^) in PBS for 30 min at 37°C. Treatment with the first antibody was performed at room temperature for 60 min with anti-cytokeratin 18 and anti-IQGAP1 antibodies, each diluted 1 : 100 in 0.2 % BSA (w v^−1^) in PBS. Afterwards, the cells were washed three times with PBS and incubated with Cy3-conjugated anti-mouse IgG diluted 1 : 2000 in 0.2% BSA (w v^−1^) in PBS for 30 min at room temperature. Nuclei were stained for 15 min at room temperature with DAPI (2 *μ*g ml^−1^ methanol). Cells were rinsed with H_2_O and the coverslips were mounted by inverting them into 10 *μ*l Mowiol (2.8 g Mowiol 4-88, Hoechst (Frankfurt am Main, Germany), 6 g glycerol, 6 ml H_2_O, 12 ml of 200 mM Tris/HCl, pH 8.5). Cells were examined with an inverse IX70 fluorescence microscope (Olympus, Hamburg, Germany) and images were recorded using a CCD camera and the analySIS 3.1 imaging system (Soft Imaging System, Münster, Germany).

## RESULTS

### MTA1 mRNA expression in pancreatic carcinoma cell lines

**Figure 1:**
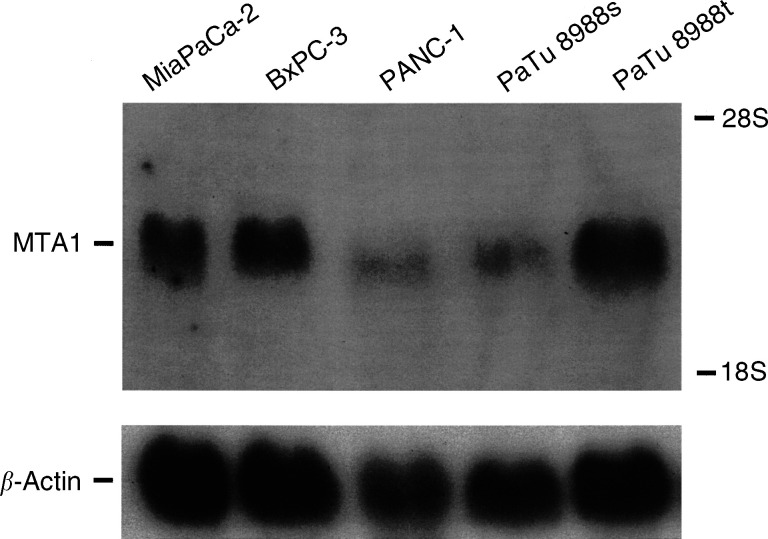
Northern blot analysis of MTA1 mRNA expression in pancreatic carcinoma cell lines. Total RNA was prepared from PANC-1, BxPC-3, MiaPaCa-2, PaTu 8988 s, and PaTu 8988t pancreatic carcinoma cells, fractionated (30 *μ*g lane^−1^) by denaturating formaldehyde–agarose (1% (w v^−1^)) gel electrophoresis and transferred onto a nylon membrane. Hybridisation was performed with a radiolabelled MTA1 cDNA probe (upper panel). To assess equal loading of the analysed RNA, the blot was rehybridised with a radiolabelled *β*-actin cDNA fragment (lower panel). The positions of the 28S and 18S ribosomal RNA bands are indicated.

To investigate the expression of MTA1 mRNA in different human pancreatic carcinoma cell lines, Northern blot analysis was performed using radiolabelled rat MTA1 cDNA for hybridisation. As shown in Figure 1

, MTA1 mRNAs of approximately 3.0 kilobases in size were present in all pancreatic carcinoma cell lines, but at different expression levels. Since PANC-1 cells express only low levels of endogenous MTA1 mRNA, we chose this cell line to investigate the effects of heterologous MTA1 expression on the cell biological properties of pancreatic carcinoma cells.

### Subcellular localisation of EGFP–MTA1 in PANC-1 cells

**Figure 2:**
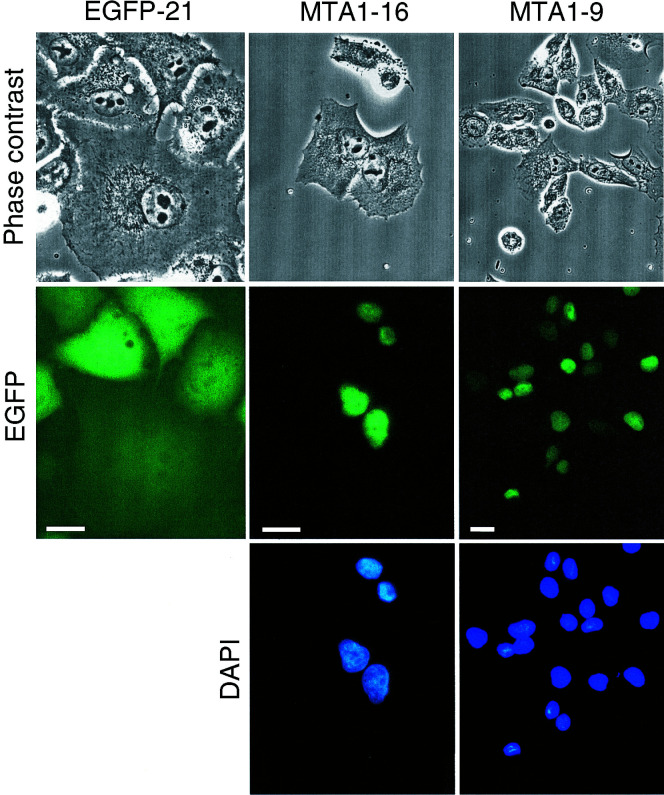
Subcellular localisation of EGFP–MTA1. Subconfluent EGFP- and EGFP–MTA1-expressing PANC-1 cells were fixed and stained with DAPI to visualise cell nuclei. Images in the top panel represent phase contrast images of PANC-1 cells expressing either EGFP alone (*EGFP-21*) or the EGFP–MTA1 fusion protein. Cells from two clones expressing EGFP–MTA1 (*MTA1-16*, *MTA1-9*) are shown. Images in the middle and bottom panels represent fluorescent micrographs of the same cells showing the localisation of EGFP and EGFP–MTA1, and DAPI-stained nuclei. Bars: 20 *μ*m.

For expression of exogenous MTA1 in PANC-1 cells, the cDNA of rat MTA1 was cloned into a pEGFP-expression vector to obtain an EGFP–MTA1 fusion protein. PANC-1 cells were transfected with the vectors pEGFP or pEGFP–MTA1 and cell clones with stable expression of the encoded proteins were selected by G418 treatment. Four of the cell clones expressing EGFP–MTA1 (named MTA1-7, MTA1-8, MTA1-9, MTA1-16) were used for further analysis. The EGFP-expressing PANC-1 cell clones EGFP-14 and EGFP-21 served as controls. Figure 2

shows the results of a fluorescence microscopical comparison of one EGFP-expressing PANC-1 clone (EGFP-21) and two EGFP–MTA1-expressing clones (MTA1-9 and MTA1-16). The images demonstrate that EGFP proteins were homogeneously distributed in the cytosol and in the nucleus, whereas EGFP–MTA1 fusion proteins showed a predominantly nuclear localisation (centre panels). The nuclear localisation of EGFP–MTA1 was verified by costaining of nuclei with 4,6-diamino-2-2-phenylindol (DAPI) (bottom panels). Similar results were obtained for the PANC-1 clones EGFP-14 and EGFP–MTA1-7 and MTA1-8 (data not shown). In additional experiments (results not shown), the localisation of MTA1 in the nucleus was further confirmed by subcellular fractionation and immunoblot analysis.

### Effects of EGFP–MTA1 on cell growth

Expression of EGFP–MTA1 in PANC-1 cells led to a significant inhibition of cell proliferation, as shown in Table 1

**Table 1:** Proliferation of PANC-1 cells expressing EGFP or EGFP–MTA1

**Cell clone**	**Doubling time (h) (±s.e.m, *n*=3)**	**Increase in doubling time (%) compared to EGFP-21 cells**
EGFP-21	37.4±1.7	—
MTA1-7	43.6±1.4	16% (*P*< 0.01, *t*-test)
MTA1-8	52.9±0.9	40% (*P*< 0.001, *t*-test)
MTA1-9	56.0±2.7	50% (*P*< 0.001, *t*-test)
MTA1-16	48.4±1.4	28% (*P*< 0.001, *t*-test)

PANC-1 cells expressing either EGFP (*EGFP-21*) or EGFP–MTA1 (*MTA1-7*, *MTA1-8*, *MTA1-9*, *MTA1-16*) were grown in DMEM supplemented with 10% FCS. Cell growth was determined by counting the cells every 48 h over a period of 12 days. Growth curves were calculated by nonlinear regression analysis for exponential growth and the average doubling times were determined using the GraphPad Prism 3.02 software. The means ±s.e.m of three independent experiments are presented. Statistical analysis was performed using the Student's *t*-test.

. The EGFP-expressing cell clone EGFP-21 exhibited the shortest doubling time, approximately 37 h, closely corresponding to the doubling time observed for the parental PANC-1 cells previously determined to be approximately 36 h (Giehl *et al*, 2000). Compared to control cells, EGFP–MTA1-expressing cells from the four independent clones, MTA1-7, MTA1-8, MTA1-9, and MTA1-16, displayed an increase in the doubling time ranging between 16 and 50%.

To determine whether substrate-independent growth of PANC-1 cells (Giehl *et al*, 2000) was altered by expression of the EGFP–MTA1 protein, soft agar assays were performed on clone MTA1-16 and the control clone EGFP-21. Determination of the average colony size at day 28 showed no difference between the two cell clones. The sizes of colonies formed by EGFP-21 control cells and EGFP–MTA1-expressing MTA1-16 cells were 219±20 and 199±9 μm (means±s.e.m, *n*=3), respectively. Since the numbers of colonies formed during the incubation time also showed no obvious differences, we conclude that overexpression of MTA1 does not alter the capacity of PANC-1 to grow in a substrate-independent manner.

### Influence of MTA1 on cell migration and invasion

**Figure 3:**
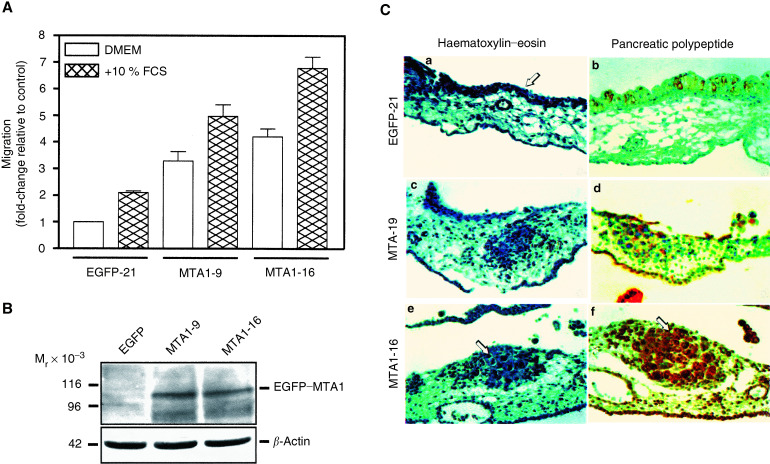
Stimulation of tumour cell migration and invasion by EGFP–MTA1. (**A**) Cell migration. PANC-1 cells (5 × 10^4^ cells well^−1^) expressing either EGFP (*EGFP-21*) or EGFP–MTA1 fusion protein *(MTA1-9* and *MTA1-16)* were seeded in trans-well cell migration inserts (8 *μ*m pore size). The upper chamber contained DMEM without supplements. The lower chamber was filled with either DMEM (empty bars) or DMEM supplemented with 10% FCS (hatched bars). The incubation was carried on for 24 h, and the cells were then fixed and stained with haematoxylin. Cells that had migrated to the bottom side of the filters were counted in three randomly selected microscopic fields (magnification: × 100) per filter. The fold-changes relative to the cell number observed for control EGFP-expressing PANC-1 cells expressing in the presence of DMEM without serum are shown as means ±s.e.m of three independent experiments. The stimulatory effect of augmented MTA1 expression on cell migration was statistically significant at a level of *P*< 0.05 both in the absence and in the presence of 10% FCS, as determined by Kruskal–Wallis one-way analysis of variance. (**B**) PANC-1 cells expressing either EGFP (*EGFP*) or EGFP–MTA1 (*MTA-9, MTA-16*) were homogenised in RIPA buffer and 130 *μ*g of protein were subjected to SDS–PAGE and immunoblotting. Immunoreactive proteins were detected using antibodies reactive against EGFP and ECL detection system. To control for equal loading, the blot was reprobed with an antibody reactive against *β*-actin. The positions of EGFP–MTA1, *β*-actin, and the molecular weight markers are shown. (**C**) Cell invasiveness. PANC-1 cells expressing either EGFP (*EGFP-21*) or the EGFP–MTA fusion protein (*MTA1-9*, *MTA1-16*) (5 × 10^5^ cells egg^−1^) were grafted onto the chorioallantoic membrane of fertilised chicken eggs on day 5 of the breeding protocol. After 6 days, the chorioallantoic membranes were removed, embedded in paraffin, and cut into 4 *μ*m sections. The sections were then either stained with haematoxylin–eosin to assess cell morphology (left panel) or subjected to immunostaining using antibodies reactive against pancreatic polypeptide to specifically identify PANC-1 pancreatic carcinoma cells (right panel). EGFP- or EGFP–MTA1-expressing PANC-1 cells are indicated by arrows. Magnification: × 200.

The migratory behaviour of EGFP- and EGFP–MTA1-expressing PANC-1 cells was analysed using trans-well migration assays. Both types of PANC-1 cells share a similar size and typically display a diameter of approximately 20 *μ*m. Cells were seeded on top of a porous membrane and migration was analysed in the absence or presence of FCS as chemoattractant in the lower compartment. Figure 3A

shows that, in the absence of FCS, expression of EGFP–MTA1 in MTA1-9 and MTA-16 cells caused an approximately 3.3- and 4.2-fold increase in cell migration, respectively, as compared to EGFP-21 cells. Addition of FCS to the lower compartment increased the number of migrating cells in control and EGFP–MTA1-expressing cells. Specifically, migration of EGFP-21 cells was increased by 2.1-fold in the presence of FCS in comparison to untreated EGFP-21 cells. In the presence of FCS, expression of EGFP–MTA1 in MTA1-9 and MTA-16 PANC-1 cells caused a 2.4-fold and 3.2-fold increase compared to the corresponding EGFP-21 cells. Thus, under both conditions, that is with and without FCS as chemoattractant, EGFP–MTA1-expressing cells exhibited an approximately three-fold enhanced cell motility as compared to controls. The immunoblots shown in Figure 3B demonstrate that both EGFP–MTA1 cell clones expressed approximately the same amount of EGFP–MTA1 protein. Thus, at least in the two cell clones analysed, there was no apparent correlation between EGFP–MTA1 expression and enhancement of motility and invasiveness.

To examine the effect of EGFP–MTA1 expression on the invasive potential of PANC-1 cells, their ability to invade through the outer epithelial cell layer of the CAM of fertilised chicken eggs into the underlying mesenchymal tissue was examined. Figure 3C depicts photomicrographs of CAM tissue sections 6 days after inoculation of EGFP-expressing EGFP-21 control cells and EGFP–MTA1-expressing MTA1-9 and MTA1-16 cells onto the CAM. Enhanced green fluorescent protein-expressing control cells were exclusively located on top of the outer epithelial membrane of the CAM and did not penetrate through this boundary (panel a, arrow). In striking contrast, cells expressing EGFP–MTA1 broke through the epithelium membrane and invaded into the underlying mesenchyme (panels c–f). In some sections, formation of solid tumours was clearly evident (panels e and f, arrows). Furthermore, the CAM was markedly thickened around this area of infiltration. To ascertain that these tumours were indeed derived from exogenous PANC-1-cells, immunostaining of the tissue with antibodies directed against pancreatic polypeptide was performed. The results confirmed that EGFP-21 control cells did not penetrate through the outer epithelium of the CAM (b, arrow), and that the tumours in the mesenchymal tissue were indeed of pancreatic origin, that is were formed by EGFP–MTA1-expressing cells.

### MTA1-induced changes in the arrangement of cytoskeletal proteins

**Figure 4:**
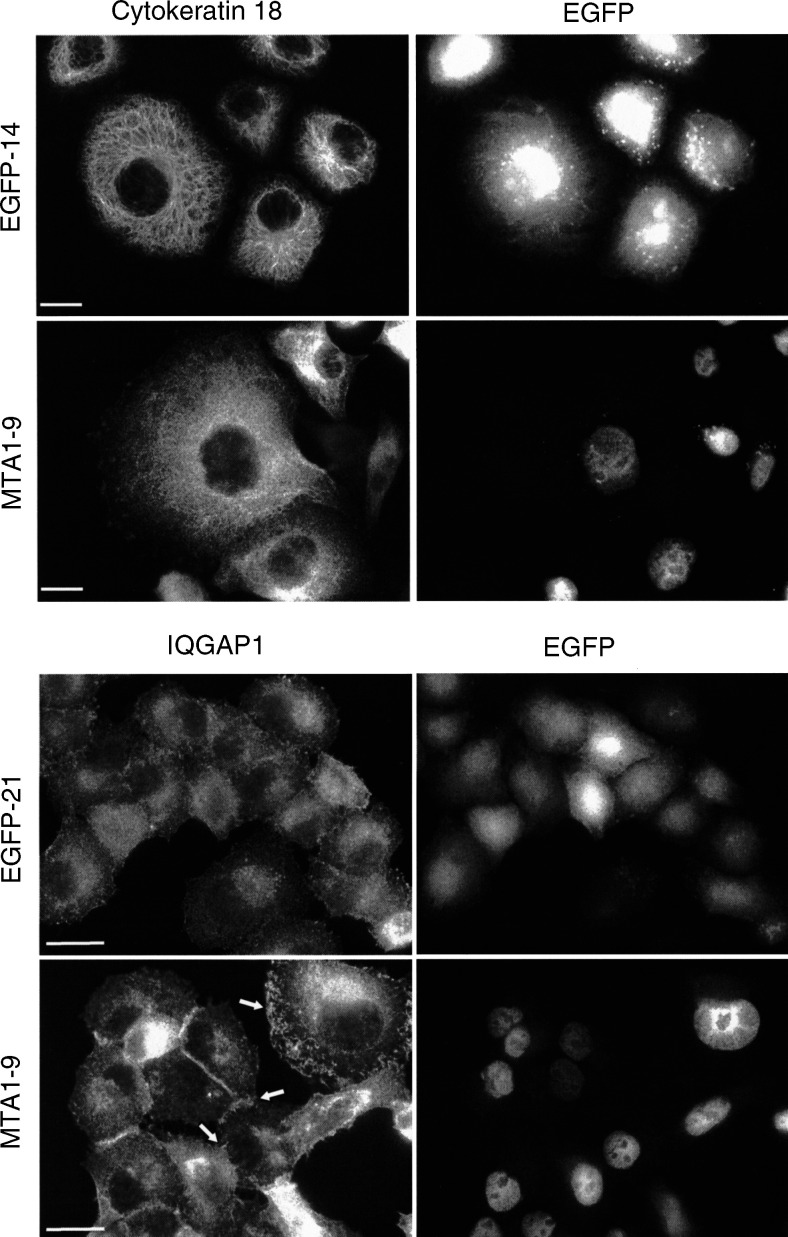
Effect of MTA1 on the organisation of the intermediate filament system and the localization of IQGAP1. Subconfluent PANC-1 cells expressing either EGFP (*EGFP-14*) or EGFP–MTA1 (*MTA1-9*) were grown for 48 h in DMEM supplemented with 10% FCS, fixed, and immunostained as indicated with antibodies reactive against cytokeratin 18 or IQGAP1 followed by Cy3-conjugated second antibodies. Enhanced green fluorescent protein fluorescence was visualised for control. Membrane ruffles and protrusions are marked by arrows. Bars: 20 *μ*m.

The acquisition of a motile, invasive phenotype is accompanied by a change in the architecture and composition of the cytoskeleton and cell–cell adhesion complexes. Previous publications have demonstrated an unexpected coexpression of the intermediate filament proteins cytokeratins and vimentin in various tumour cells, which seems to correlate with their metastatic potential (Hendrix *et al*, 1996; Thomas *et al*, 1999). To address the question whether expression of EGFP–MTA1 altered the expression of these proteins, immunoblot analyses were performed. Both cytokeratins and vimentin were found to be expressed in parental PANC-1 cells and the total amount of these proteins did not change upon expression of EGFP or EGFP–MTA1 (data not shown). To examine whether expression of EGFP–MTA1 affects the subcellular distribution and organisation of cytokeratin filaments, immunofluorescence analyses were performed on EGFP-14 control cells and MTA1-9 cells using an antiserum reactive against human cytokeratin 18 (Figure 4

, top panels). The results revealed that the extensive filamentous network composed of cytokeratin 18-containing filaments was much more diffuse, and that the long filaments seemed to be dissolved in MTA1-expressing cells as compared to EGFP-expressing controls. Quantitative analysis revealed that only 20% of EGFP–MTA1-expressing PANC-1 cells exhibited a well-organised cytokeratin filament system, while approximately 80% of EGFP-expressing or parental PANC-1 cells exhibited a well-organised cytokeratin filament system (data not shown). These changes were not only apparent upon staining of cells with a cytokeratin 18, but were also evident after staining with a pankeratin antibody. The organisation of vimentin filaments was not markedly altered (data not shown).

Another process involved in cell migration is the dynamic rearrangement of cell–cell adhesion complexes and the actin cytoskeleton. Localised disruption of E-cadherin-mediated cell–cell adhesion sites was recently observed in invasive human colon carcinoma cells (Shimao *et al*, 2002) and this disruption was associated with relocalisation of IQGAP1, a Rac1/Cdc42 effector molecule (Kuroda *et al*, 1998). Therefore, the subcellular distribution of IQGAP1 was studied by immunofluorescence analysis (Figure 4, bottom panels). The results showed a marked increase in membrane-localised IQGAP1 and a decrease in the cytoplasmic IQGAP1 immunostaining in EGFP–MTA1-expressing MTA1-9 cells in comparison to EGFP-expressing EGFP-21 control cells. Furthermore, IQGAP1 seemed to be enriched in membrane ruffles and protrusions.

## DISCUSSION

In the present study, we have investigated the functional consequences of stable heterologous expression of an EGFP–MTA1 fusion protein in the human pancreatic carcinoma cell line PANC-1. The results presented here demonstrate that expression of EGFP–MTA1 (i) resulted in a nuclear localisation of the protein, (ii) caused an attenuation of cell proliferation, but (iii) markedly increased cell migration and invasion, and (iv) induced distinct changes in the organisation of the cytokeratin filament system and the localisation of cytoskeleton-associated proteins.

The nuclear localisation of EGFP–MTA1 in PANC-1 cells is in agreement with the domain structure of MTA1 comprising a BAH and a SANT domain, which have been found in proteins involved in transcriptional regulation (Aasland *et al*, 1996; Callebaut *et al*, 1999). As shown in Table 1, expression of EGFP–MTA1 fusion protein decreased the proliferation rate of PANC-1 pancreatic carcinoma cells. This observation is in contrast to results describing growth inhibition of MDA-MB-231 breast cancer cells upon treatment with MTA1 antisense oligonucleotides (Nawa *et al*, 2000). However, neither overexpression of MTA1 nor inhibition of MTA1 expression had any detectable effect on cell cycle progression or proliferation of HaCaT keratinocytes (Mahoney *et al*, 2002), MCF-7 (Mazumdar *et al*, 2001), or MDA-MB-435 breast cancer cells (Nawa *et al*, 2000). The reasons for these divergent findings are currently unclear, but might be related to differences in growth-regulating signalling pathways that are either unrelated to or less dependent on MTA1 expression. Although EGFP–MTA1-expressing PANC-1 cells proliferated slower when cultured on plastic, the substrate-independent growth of PANC-1 cells was not markedly affected by the expression of EGFP–MTA1. This could either mean that substrate-independent growth of PANC-1 cells is independent of MTA-1 expression or that any attenuation of this type of proliferation is compensated for by another, growth-promoting mechanism. In contrast to the results obtained here, stable expression of MTA1 in MCF-7 breast cancer cells resulted in a markedly enhanced ability of these cells to grow substrate-independently and to form large colonies in soft agar (Mazumdar *et al*, 2001). While the reasons of this apparent discrepancy are currently unknown, it seems clear that an increase in cell proliferation is unlikely to be the basis of the enhanced metastatic potential of pancreatic cancer cells with elevated MTA1 expression described by Iguchi *et al* (2000).

Metastatic cells exhibit an enhanced rate of cell migration, an enhanced ability to penetrate epithelial layers, and an enhanced tendency for invasive growth (Wells, 2000). The results of this study show that cells expressing EGFP–MTA1 showed a markedly enhanced cell motility. In addition, serum components acting as chemoattractants further stimulated cell migration of EGFP–MTA1-expressing PANC-1 cells, suggesting that the expression of MTA1 accelerates the overall motility of PANC-1 cells. This enhanced motile response was even more evident when the ability to penetrate into a three-dimensional tissue was analysed. Thus, only the EGFP–MTA1-expressing PANC-1 cells broke through the CAM and formed solid tumours in the mesenchymal tissue of the CAM. These results show that EGFP–MTA1-expressing PANC-1 cells have acquired the ability to penetrate an epithelial layer and to successfully prevail against CAM cells in the underlying tissue. Iguchi *et al* (2000) investigated the correlation between MTA1 gene expression and metastatic potential of pancreatic cancer in patients and of pancreatic cancer cell lines. Although MTA1 mRNA expression did not correlate with the metastatic potential in the analysed pancreatic carcinoma cell lines, as evaluated by a liver metastasis assay in nude mice, MTA1 mRNA expression levels in pancreatic cancer tissues seemed to be correlated with the occurrence of lymph node metastasis in the corresponding patients. On the basis of their results, Iguchi *et al* suggest a possible role for MTA1 in the progression of pancreatic cancer, in particular with regard to giving rise to lymph node metastases. Furthermore, a correlation between MTA1-expression, invasion, and migration has recently been shown for immortalised keratinocytes (Mahoney *et al*, 2002). Taken together, results in the literature and our findings demonstrate that elevated MTA1 expression augments the capacity of epithelial cells for metastatic dissemination.

How MTA1 promotes the more motile, invasive phenotype of PANC-1 pancreatic carcinoma cells is currently unknown. Modulation of cell shape by remodelling of the cytoskeleton and alterations of cell surface adhesion molecules are prerequisites for invasive growth (Wells, 2000). We could not detect any obvious changes in the organisation of the tubulin or actin filament system in adherent EGFP–MTA1-expressing cells (data not shown). Interestingly, the cytoskeleton-associated protein IQGAP1 was predominantly localised at the membrane of EGFP–MTA1-expressing cells, whereas it showed a cytoplasmatic localisation in the EGFP-expressing control cells. Membrane localisation of IQGAP1 was previously found to be inversely correlated with the degree of differentiation of gastric carcinoma cells (Takemoto *et al*, 2001) and was observed in human colorectal carcinoma cells, especially at the invasion front (Nabeshima *et al*, 2002), as well as in human colon carcinoma cells following treatment with heptocyte growth factor (HGF) (Shimao *et al*, 2002). IQGAP1 is an actin- and Cdc42-binding protein (Erickson *et al*, 1997; Swart-Mataraza *et al*, 2002) and it has also been shown to negatively regulate the E-cadherin/catenin-based cell–cell adhesion by dissociating *α*-catenin from the E-cadherin/catenin complex (Fukata *et al*, 1999; Kuroda *et al*, 1998). Thus, translocation of IQGAP1 to the plasma membrane and reduced cell–cell adhesion as a consequence of heterologous EGFP–MTA1 expression might be a reason for the observed enhanced cell motility and invasion.

In addition to IQGAP1 translocation, MTA1 induced changes in the organisation of the cytokeratin system. PANC-1 cells express different cytokeratins, such as the acidic cytokeratins 18 and 19 and the basic cytokeratin 8 (data not shown). These cytokeratins are also found in ductal adenocarcinoma of the pancreas (Hoorens *et al*, 1998). Expression of EGFP–MTA1 caused dissolution of the distinctively structured filamentous cytokeratin network, giving rise to a network of filiform bundles as well as aggregates at the cell periphery (cf. Figure 4). Although cytokeratin filaments have been considered to be relatively stable cytoskeletal structures, there is increasing evidence for dynamic processes in cell migration, cytokinesis, and apotosis (Goldman *et al*, 1999; Strnad *et al*, 2002, and literature cited therein). Very little is known about the molecular principles that govern the dynamics of cytokeratin filament organisation, but phosphorylation of cytokeratins at serine and threonine residues causes major rearrangements of the filament structure in various cell types (Herrmann and Aebi, 2000; Strnad *et al*, 2002). How EGFP–MTA1 remodels the cytokeratin filament system in PANC-1 cells remains to be determined.

In summary, this study demonstrates that heterologous expression of EGFP–MTA1 facilitates the acquisition of an invasive, metastatic phenotype of PANC-1 pancreatic carcinoma cells. Enhanced expression of MTA1 might be of considerable importance for the progression to malignancy of pancreatic neoplasms.
